# Designing an educational intervention on second-hand smoke in smoker men on the exposure of pregnant wives: a protocol for a randomized controlled trial

**DOI:** 10.1186/s12978-019-0673-1

**Published:** 2019-02-01

**Authors:** Zohreh Karimiankakolaki, Seyed Saeed Mazloomy Mahmoodabad, Ashraf Kazemi, Hossein Fallahzadeh

**Affiliations:** 1 Social Determinants of Health Research Center, Department Of Health Education And Promotion, School Of Public Health, Shahid Sadoughi University Of Medical Sciences, Yazd, Iran; 2 Social Determinants of Health Research Center, Department Of Health Education And Promotion, School Of Public Health, Shahid Sadoughi University Of Medical Sciences, Yazd, Iran; 3 Department of Reproductive Health, School of Nursing and Midwifery, Isfahan University Of Medical Sciences, Isfahan, Iran; 4 Department of Biostatistics and Epidemiology, Shahid Sadoughi University Of Medical Sciences, Yazd, Iran

**Keywords:** Educational intervention, Secondhand smoke, Smoker men, Pregnant women

## Abstract

**Background:**

Complications of exposure to second-hand smoke (SHS) are known to affect the health of pregnant women; it requires designing an educational program to reduce the exposure of pregnant women to smoke. Therefore, the purpose of this study was to design an educational program to reduce the exposure of pregnant women with the second-hand smoke of their husband.

**Methods:**

This research-based program is designed in three phases, in the first phase of the study, the level of knowledge, attitude, and practice of men regarding SHS will be measured using a questionnaire. The questionnaire will be based on a literature review and experts opinions. In the next phase the educational program will be designed based on the results of the first phase of the study on the level of knowledge, attitude, self-efficacy, and practice of men, as well as a literature review and experts opinions, and the research team will finalize it according to priorities. To follow up the training will be sent reminders messages. Pregnant women will also be trained to protect themselves and support their husbands to quit smoking. The third phase includes the implementation of educational intervention with pretest-posttest with two intervention and control groups.

**Discussion:**

The present study provides strong information and data regarding the needs and strategies for reducing the exposure to second-hand smoke in pregnant women. Therefore, designing a program, and a review article and updated evidences can lead to avoid pregnant women the exposure to second hand smoke and reducing smoking in smoker husbands. It can also reduce their medical and treatment costs.

**Trial registration:**

Registration of this randomized control trial has been completed with the Iranian Registry of Clinical Trials, IRCT20180722040555N1.

## Plain English summary

Second-hand smoke exposure is a risk factor for the health of pregnant women, and associated with dangers such as preterm labor, rupture of membranes, cesarean delivery, decreased growth, and intrauterine growth retardation, fetal weight loss, and so on. The exposure to smoke is including breathe of smoke from burning cigarette and smoke from exhaling of smokers. Although the prevalence of female smokers is low in Iran, the high prevalence of smoking among men put women at the risk of SHS. The aim of this study was to design an educational intervention on the smoke of SHS in smoker men on the exposure of pregnant wives. This research-based program is designed in three phases, in the first phase of the study; the level of knowledge, attitude, and practice of men regarding SHS will be measured using a questionnaire. The questionnaire will be based on a literature review and experts opinions. In the next phase the educational program will be designed based on the results of the first phase of the study on the level of knowledge, attitude, self-efficacy, and practice of men, as well as a literature review and experts opinions, and the research team will finalize it according to priorities. To follow up the training will be sent reminders messages. Pregnant women will also be trained to protect themselves and support their husbands to quit smoking. The third phase includes the implementation of educational intervention with pretest-posttest with two intervention and control groups. In the pretest phase, questionnaires will be completed by men and the exposure of women to SHS will be achieved as self-reported. Two months after the training in the post-test phase, the questionnaire will be completed again by men. The exposure of pregnant women to SHS and the amount of salivary cotinine will be measured.

## Background

Smoking is an important risk factor for health and development [1]. Smoking habits are seen among various social groups so that about 50% of men in developing countries smoke and 35% in developed countries [2]. The exposure to smoke is including breathe of smoke from burning cigarette and smoke from exhaling of smokers ([3], https://www.cdc.gov/tobacco/data_statistics/fact_sheets/secondhand_smoke/general_facts/). In the world, more than 40% of children, 33% of non-smokers men and 35% of non-smokers women are exposed to SHS [4–6]. Predicted, by 2030 the death rate from tobacco will be more than 8 million, which approximately 5.2 million are women [2]. Although the prevalence of female smokers is low in Iran, the high prevalence of smoking among men put women at the risk of SHS [7], more than half of Iranian women, during pregnancy (56.2%) were exposed to second-hand smoke [8]. Exposure to SHS can lead to ischemic heart disease, respiratory infections, asthma, and lung cancer [4]. Complications, especially in pregnant women, are significant, such as preterm labor [9–13], rupture of membranes [14], cesarean delivery [10], decreased growth, and intrauterine growth retardation [2, 9, 13], low birth weight fetus [9, 11–13], distress fetus [11, 12], small fetus compared to gestational age [10], Sudden infant death syndrome and increasing the Cotinine level in follicular fluid in women were exposed to SHS [15]. Husband’s smoking is a source of tobacco smoke exposure at home [16]. The only way to protect non-smokers is to reduce smoking in the home, the work environment, and the general environment, and the highest exposure to SHS occurs at home (https://www.cdc.gov/tobacco/data_statistics/fact_sheets/secondhand_smoke/general_facts/). Effective interventions to reduce exposure to SHS in pregnant women are necessary and the content of these interventions should be for both pregnant women and their husbands [6]. Wakefield reported that creating messages that raise awareness and acceptance of the risks of exposure to SHS in pregnancy and childhood, may increase the incentive of men to quit smoking and that encouraging men to participate and support their wife during pregnancy, finally will lead them to quit smoking [17]. Educational interventions for smoking could reduce smoking rates for men during the pregnancy of his wife; therefore, pregnancy can be a motivating factor for changing the behavior of smoker men [18], and husbands’ awareness of the risks and prenatal care is effective in contributing to the health of pregnant women [19]. Therefore, interventions should be designed, in addition to raising knowledge and changing the attitude and practice of smoker husbands towards cigarette smoking, can reduce exposure to SHS in pregnant women. The purpose of this study was to design an educational program about SHS in smoker men on the exposure of their pregnant wives.

## Methods/design

This research-based program is designed in three phases, in the first phase of the study; the level of knowledge, attitude, and practice of men regarding SHS will be measured using a questionnaire. The questionnaire will be based on a literature review and experts opinions. In the next phase the educational program will be designed based on the results of the first phase of the study on the level of knowledge, attitude, self-efficacy, and practice of men, as well as a literature review and experts opinions, and the research team will finalize it according to priorities. To follow up the training will be sent reminders messages. Pregnant women will also be trained to protect themselves and support their husbands to quit smoking. The third phase includes the implementation of educational intervention with pretest-posttest with two intervention and control groups.

## Aim

In this study, we are looking at designing an educational program on SHS in the smoker men, in addition to raising knowledge and changing the attitude and practice of smoker husbands towards cigarette smoking, can reduce exposure to SHS in pregnant women.

## Research hypotheses

According to the main purpose of the study, hypotheses will be considered according to the views of health education and reproductive health professionals. The mean score of knowledge, attitude, and practice of smokers after intervention in the intervention group will be more than the control group. The mean of salivary cotinine and the average number of cigarettes that exposed to them during one week in pregnant women after intervention in the intervention group will be less than the control group.

### Phase I: Designing a questionnaire on knowledge, attitude and practice of smoker men

First, a literature review will be done to select the appropriate model, after interviewing the panel of health education professionals, promotion of health, psychology, epidemiology and reproductive health and reviewing the studies, the questionnaires will be designed based on some of the constructs of the health belief model. The questionnaire consists of the following structures: knowledge, perceived sensitivity, perceived severity, self-efficacy, and behavior. To assess the validity of the questionnaire, a fifteen-person panel of health education professionals, health promotion, psychology, epidemiology and reproductive health will be used and CVR and CVI will be obtained to verify structures. To verify the reliability of the questionnaire, internal consistency with alpha Cronbach will be measured. The questionnaire will be used in the pre-test and post-test of the intervention phase.

### Phase II: Designing an educational intervention

The design of the intervention and the target audience’s recognition, at this stage, will be based on a review of the literature, studying the similar studies, as well as information from the questionnaire designed in the previous phase. In this study, the educational package with regard to time limitation of contacts includes lecture sessions (30–60 min), educational pamphlets, animations about second-hand smoke harms, fetus photos, reminiscent text messages and the training of pregnant women to support their husband for reducing smoking. Types of educational messages are listed in Table 1.

**Table 1:** Types of educational package messages

*• Educational messages*: Content about cigarette smoke, SHS and its complications presented at a lecture and pamphlet (e.g. SHS causes preterm labor).	
**•** *Functional messages*: Describe the simple steps to quit smoking and protect your wife from smoke presented at a lecture and pamphlet (e.g. put lighter and ashtray away from your reach).	
**•** *Motivational messages*: Includes images and animations (examples: images included in the pamphlets and animations related to cigarettes and its effects).	
**•** *Self-efficacy promotion messages*: Breaking complex behaviors into simple steps, presented in Pamphlet and use the role model at a lecture session (e.g. Simple steps to quit smoking and protective strategies, key sentences about the ability to quit smoking, expressing the experiences of a smoker from the history of smoking cessation and having the ability to quit smoking).	
**•** *Alert or reminder messages*: Message or content to remind you of second-hand smoke complications for pregnancy and embryos (including a picture will be given to the participants to sit at their home in front of their eyes; this photo is “a fetus who asks the father not to smoke” and sending reminders).	
**•** *Supportive messages*: Getting help from pregnant women as “educational co-sponsors” to support their husbands to quit smoking.	

#### Training session (lecture, pamphlets, and expressions of experiences of people with a history of smoking cessation)

The lecture will contain information on pregnancy and childbirth, cigarette smoke, and the diagnosis of all types of smoke and second-hand smoke complication for pregnancy and fetuses and recommendations for the protection against smoke. It will be presented as a lecture to raise knowledge and attitudes. Animation, educational pictures and educational pamphlets will be used to motivate. Simple steps to stop smoking and to express the experiences of a smoker, who has previously had a history of cessation of smoking, will be used to increase self-efficacy.

#### Follow up and maintaining education (fetus photo, SMS reminder, receiving support from pregnant women)

At the end of the lecture, a picture will be given to the participants to sit at their home in front of their eyes; this photo is “a fetus who asks the father not to smoke”. After training, for two months at the end of each week, an alert SMS will be sent to the participants. Pregnant women will be trained to protect against second smoke and support their husbands to quit smoking. Pregnant women will be as “educational co-sponsors”.

## Experts’ team

The content and visual validity of the questionnaire and educational program will be measured, and the comments received from the panel of experts. The panel of experts will be included eight professionals from health education, one expert from the health promotion field, one expert from the epidemiology, four professionals from the reproductive health and one expert from the field of psychology.

### Phase III: Implementation of educational intervention

In this step a randomized controlled clinical trial will design to assess the efficacy of prepared protocol. Participants randomly will allocate into two groups. In experimental group we will implement our designed protocol, and control group will not receive any intervention, but they follow as same as experimental group. For ethical issues all educational material will offer them at the end of study.

#### Study environment and population

This interventional randomized study will be conducted at health centers of Isfahan, Iran.

#### Study sample

This interventional randomized study will be conducted on smoking men who have exposed their pregnant wives to cigarette’s smoke and their wives have been receiving maternity care at health centers of Isfahan, Iran.

#### Sampling method

Convenience sampling’s method will apply to select the participants. The sampling method will be random and the participants will be in two groups of intervention and control.

#### Inclusion criteria

Inclusion criteria are smoker men with pregnant women aged between 15 and 49 years, the men who smoke at least one cigarette daily beside their pregnant wives, Iranian nationality, consent to participate in the study, ability to understand questions or ability to read and write, not participating in any other clinical trials at the same time.

#### Exclusion criteria

The exclusion criterion is not completing the intervention for any reason, the smoker men who are not with their pregnant wives for more than one week, the pregnant termination, and the death of the spouse or immigration and active smoker women and presence of other sources of exposure such as other smokers at home, smoke exposure at work, women’s active smoking, etc.

#### Data collection method

The instrument for collecting mental health data is the researcher-made questionnaire about smoker men’s knowledge, attitude and practice regarding to second hand smoke in pregnancy. This questionnaire has subscales including knowledge, attitude and practice. Pregnant women will be asked about exposure to SHS before and after intervention. Saline Cotinine will be measured by pregnant women after the intervention using a saliva kit.

##### Data analysis

We will apply X2 and independent T test to analysis our data by using SPSS version 21.

#### Outcome measures

##### Knowledge, attitude and practice of smoker men

The mean score of knowledge, attitude and practice of smoker men will be measured using a researcher-made questionnaire in the pre-test and post test phase.

#### Self-reported of pregnant women of exposure to second-hand smoke

Pregnant women will be questioned about the number of cigarettes that have exposed with them for one week.

##### Salivary cotinine pregnant women

Saliva Pregnant women will be collected and frozen by special containers and will be transferred to the lab by maintaining a cold chain, Then using the ELISA test measure cotinine basis and will be reported.

## Discussion

Various studies have shown, creating messages that raise awareness and acceptance about the dangers of the second hand smoke in pregnancy and childhood can increase men’s incentive to quit smoking, and that encouraging men to participate and support their wives in prenatal care will push them to quit smoking [17–20]. Another study showed that it could not be expected that with brief intervention alone, exposure to SHS was reduced, rather, to reduce exposure, strategies should be employed to involve parents and other family members in order to have more impact [9]. Evaluation of the exposure to cigarette smoke and its avoidance strategies is a crucial part of the pregnancy care programs [12]. The content of educational interventions should be for both pregnant women and their husbands, and the use of community-based and theory-based interventions creates a framework for programs and attempts to change the smoking culture around pregnant women [6].

The present study provides strong information and data regarding the needs and strategies for reducing the exposure to second hand smoke in pregnant women. Therefore, designing a program, and a review article and updated evidences can lead to avoid pregnant women the exposure to second hand smoke and reducing smoking in smoker men. It can also reduce their medical and treatment costs.

We suppose this program has capacity to integrate into the professionally health care guidelines, so that it can help medical and health care providers pay attention to the important role of the women health, especially during gestational age. The strategies of this program could be important and cost effective, and therefore we hope that the success of such a program is a step forward in improving their health status and men’s participation in pregnant women’s health care programs In designing the present intervention, in addition to training men, training pregnant women will be used to protect themselves as well as to help train smokers to quit smoking. The strength of this intervention was the use of the cotinine index, along with the self-reporting of exposure to cigarette smoke to measure exposure levels.

## Abbreviation

SHS: Second-hand Smoke
